# Investigation of the binding and cleavage characteristics of N1 neuraminidases from avian, seasonal, and pandemic influenza viruses using saturation transfer difference nuclear magnetic resonance

**DOI:** 10.1111/irv.12184

**Published:** 2013-09-30

**Authors:** Jean-Michel Garcia, Jimmy C C Lai, Thomas Haselhorst, Ka Tim Choy, Hui-Ling Yen, Joseph S M Peiris, Mark von Itzstein, John M Nicholls

**Affiliations:** a HKU-Pasteur Research CentreHong Kong SAR, China; b Department of Pathology, University of Hong KongHong Kong SAR, China; c Institute for Glycomics, Gold Coast Campus, Griffith UniversityGold Coast, Qld, Australia; d School of Public Health, University of Hong KongHong Kong SAR, China

**Keywords:** Epitope mapping, influenza, neuraminidase, nuclear magnetic resonance, receptor interaction, saturation transfer difference

## Abstract

**Objectives:**

The main function of influenza neuraminidase (NA) involves enzymatic cleavage of sialic acid from the surface of host cells resulting in the release of the newly produced virions from infected cells, as well as aiding the movement of virions through sialylated mucus present in the respiratory tract. However, there has previously been little information on the binding affinity of different forms of sialylated glycan with NA. Our objectives were then to investigate both sialic acid binding and cleavage of neuraminidase at an atomic resolution level.

**Design:**

Nuclear magnetic resonance (NMR) spectroscopy was used to investigate pH and temperature effects on binding and cleavage as well as to interrogate the selectivity of human-like or avian-like receptors for influenza neuraminidase N1 derived from a range of different influenza virus strains including human seasonal H1N1, H1N1pdm09 and avian H5N1.

**Results:**

We demonstrated that an acidic pH and physiological temperature are required for efficient NA enzymatic activity; however a change in the pH had a minimum effect on the NA-sialic acid binding affinity. Our data comparing α-2,3- and α-2,6-sialyllactose indicated that the variation in neuraminidase activity on different ligands correlated with a change in binding affinity. Epitope mapping of the sialylglycans interacting with NAs from different viral origin showed different binding profiles suggesting that different binding conformations were adopted.

**Conclusions:**

The data presented in this study demonstrated that physicochemical conditions (pH in particular) could affect the NA enzymatic activity with minor effect on ligand binding. NA cleavage specificity seemed to be associated with a difference in binding affinity to different ligands, suggesting a relationship between the two events. These findings have implications regarding the replication cycle of influenza infection in the host where different sialidase activities would influence penetration through the respiratory mucin barrier and the release of the newly generated virus from the infected cells.

## Introduction

Influenza is an acute viral infection affecting humans, domestic animals (such as poultry, pigs, horses, dogs) as well as wildlife, especially waterfowl.^1^ This complex and dynamic multispecies ecosystem results in outbreaks with a range of frequency, geographical distribution, and disease severity in the form of annual epidemics or occasional pandemics as well as sporadic zoonotic infections (H5N1, H7N7, and H9N2), ranging from mild disease to severe illness and deaths. These occurrences may lead to an increased burden on economy, social structures, and public health.^2^ Influenza is an enveloped virus of the *orthomyxoviridae* family and contains on its surface three membrane-bound proteins: hemagglutinin (HA), neuraminidase (NA), and proton channel matrix protein 2 (M2). There are three types of influenza (A, B, and C) that are able to infect humans: influenza A and B are responsible for recurrent epidemics, influenza A causes occasional pandemics, and influenza C does not cause clinically significant disease. Influenza A viruses have been classified into subtypes on the basis of the antigenic diversity of their HA (H1 to H16) and NA (N1 to N9).^3^ The recent discovery of H17N10 in bats has extended this genetic diversity although structural and functional features suggest a distinct lineage to the other HAs and NAs.^4^,^5^ HA is a trimeric elongated protein, composed of a globular head capping a stalk. It is involved in the initial stage of the infection by binding to sialylated receptors on the host cell surface, triggering the virus internalization through clathrin-mediated endocytosis. After conformational change of HA due to the low-pH environment in the endosomes, fusion of the viral and host membranes leads to the release of the genome in the cytoplasm.^6^ NA is a mushroom-shaped tetrameric protein consisting of four identical subunits each containing an enzymatic active site.^7^ The catalytic site recognizes terminal sialic acids linked to the glycoproteins or glycolipids on the cell surface and cleaves the α-ketosidic linkage. NA has previously been found to be crucially important in the influenza infection cycle in several ways. The most significant role of the protein is to release the progeny viruses from the host cell by removing the sialic acids on both infected cell surface and the virus itself. In the absence of NA, progeny viruses self-aggregate and are retained on the host cell surface. NA is also found to be important for the virus to evade the “decoy” receptors found in airway mucins that are rich in sialylglycans containing α-2,3 linkage, thereby allowing the virus to penetrate the mucus barrier. Investigation of the initial stage of viral infection using NA inhibitors has also suggested that NA would aid in viral entry.^8^ Recently, it has been shown that NA alone can enable the formation of virus-like particles (NA-VLP) and therefore may be implicated also in the release of the virus by providing an additional driving force for virus budding.^9^

In addition to the cleavage activity, it has been reported that some NA (N9 and N1) could possess hemagglutination activity associated with a second binding site distinct from the catalytic enzymatic site.^10^,^11^ This hemadsorption activity can be conferred to an NA that does not normally have this secondary binding site by mutation of amino acids in the two loops at positions 368–370 and 399–403 of NA.^12^ Crystallographic data have shown conformational similarities in the bound sialic acid between this site and the HA binding site.^13^ Initially, it was believed that the neuraminidase hemadsorption activity was conserved only in avian influenza A viruses based on sequence analysis.^14^ However, computer simulation studies suggested that key structural features of the secondary sialic acid binding site may be retained in human or swine viruses.^15^ Recently, we utilized saturation transfer difference nuclear magnetic resonance (STD-NMR) spectroscopy to investigate the binding of sialylglycans with NA, similar to a previous study with HA,^16^ and to demonstrate the role of these secondary sialic acid binding sites in influenza viruses from different origins.^17^

In the present study, NMR spectroscopy has been used to monitor the binding and cleavage of sialoglycosides at an atomic resolution. As an NA source, we have produced influenza virus-like particles (VLPs) containing N1 from highly pathogenic avian influenza H5N1 virus (N1_av_), human seasonal H1N1 virus (N1_sea_), and H1N1pdm09 virus (N1_pdm_). The effect of pH and temperature on the NA–sialic acid interactions as well as the substrate specificity between α-2,3/α-2,6 linked sialyllactoses was investigated. In addition, epitope mapping of sialyllactose interacting with the three mentioned NAs was performed.

## Results

Corresponding *Material and Methods* section can be found in supporting information (Data S1).

### Effect of temperature and pH on NA interactions with sialylglycans

**Figure 1:**
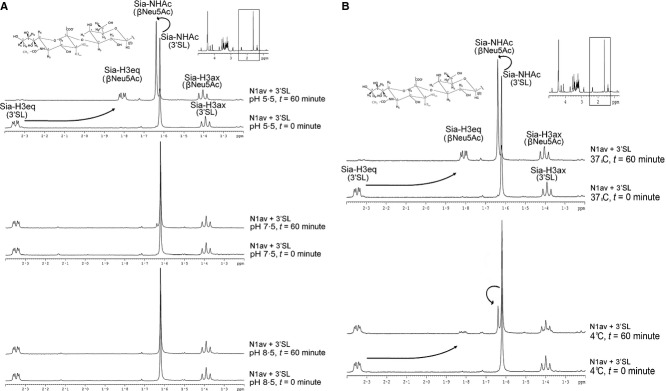
Effect of pH and temperature on cleavage activity. NA from H5N1 was incubated at different pHs (A) or temperatures (B) with α-2,3-sialyllactose. The cleavage reaction was monitored by ^1^H NMR over 1 hour.

We initially investigated the effect of temperature and pH on both the cleavage and binding using ^1^H-NMR and STD-NMR, respectively. The α-ketosidic linkage of the ligand was cleaved by NA, and the modified chemical environment of the ligand led to a change in chemical shift of the protons, which was detectable in NMR spectroscopy. Among the different changes in NMR spectra, the methyl group of N-acetamido (NHAc) and the protons carried by the carbon 3 of sialic acid (H3_eq_ and H3_ax_) were the most obvious and therefore can be used in the monitoring of NA activity. During the sialic acid cleavage, the bound form of the equatorial protons (Sia-H3_eq_) at 2·35 ppm shifted upfield to its free form at 1·82 ppm, the corresponding axial geminal proton (Sia-H3_ax_) shifted 0·01 ppm downfield, and the NHAc group had a 0·06 ppm shift downfield (as indicated by arrows in Figure 1A). The yield of cleavage can be quantified based on the Sia-H3 and Sia-NHAc signals of the 3′ sialyllactose (3′SL) comparing to those signals of the released Neu5Ac residue. After 1-hour incubation at pH 5·5, the majority (>90%) of the 3′SL was utilized, indicating that the neuraminidase was highly active. In contrast, in a neutral or mild alkaline conditions, only weak (<5%) activity (pH = 7·5) or no significant activity (pH = 8·5) was identified (Figure 1A). As expected, the sialic acid cleavage reaction at pH 5·5 was significantly reduced when a lower incubation temperature was used (from 90% at 37°C to 18% at 4°C, Figure 1B). Similar effects of pH on NA activity were obtained from N1_pdm_ and N1_sea_ (Figure S1), while the effect of temperature was only tested for N1_av_.

**Figure 2:**
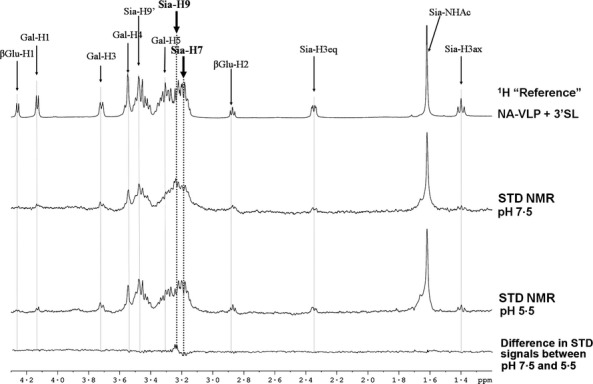
Effect of pH on binding activity of NA. NA from H5N1 was suspended at acidic (pH = 5·5) and relatively neutral pH (pH = 7·5) and incubated with α-2,3-sialyllactose. The binding of the ligand on NA was monitored by STD-NMR.

The effect of pH on the binding interaction between sialyllactose and NA-VLP was investigated using STD-NMR spectroscopy as described previously.^16^,^17^ STD-NMR signals were detected at both pH 5·5 and pH 7·5 (Figure 2). During the STD-NMR experiment, cleavage of the sialyllactose did occur, but was significantly reduced by more than 80% due to the low temperature (4°C), as indicated by the signal of cleavage in Figure 1B. A shift from acidic to neutral pH marginally reduced the overall STD-NMR signal intensity (<10%), which indicated that the change in pH had a minor effect on the binding event occurring in the substrate hydrolysis mechanism. Looking at individual signals in the two STD-NMR spectra measured at acidic or neutral pH, the only significant difference that could be identified was a reduction in signal at ˜3·18 ppm and an increment in signal at ˜3·23 ppm. These signals could be assigned to the hydrogen atom linking to the C7 and C9 of sialic acid moiety, respectively (Sia-H7 and Sia-H9, highlighted in Figure 2), probably indicating the effect of pH on specific hydrogen bonding.

### Specificity of the binding and cleavage of NA

**Figure 3:**
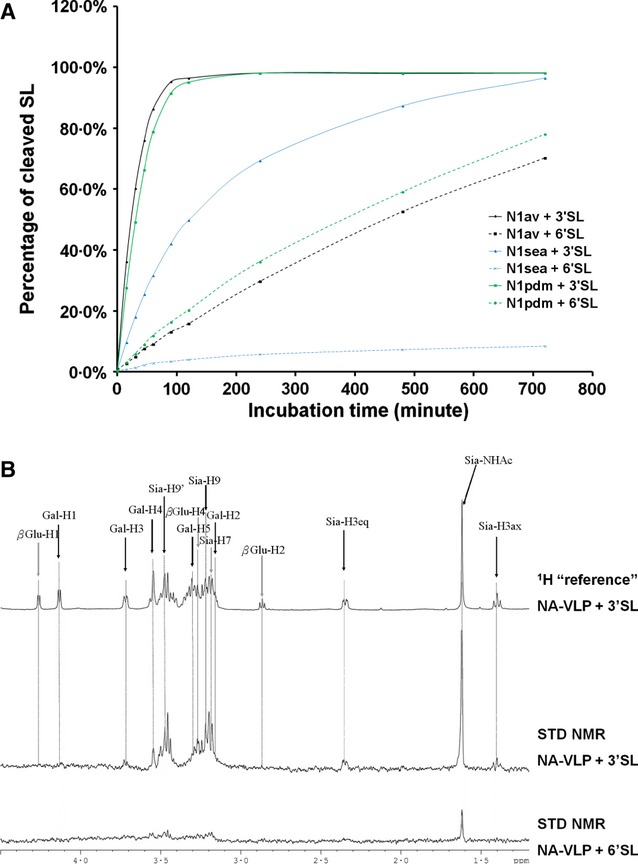
Substrate specificity of NA-VLPs. N1 from H5N1 was incubated with either α-2,3- or α-2,6-sialyllactose. The cleavage (A) and the binding (B) of the NA were monitored by ^1^H and STD-NMR, respectively. Panel B shows the N1_av_, but comparable data were obtained for N1_sea_ and N1_pdm_ (data not shown).

To evaluate the potential specificity of neuraminidase both in its enzymatic cleavage and binding, ^1^H and STD-NMR analyses of sialyllactose (3′SL and 6′SL) in complex with N1_av_, N1_sea_, and N1_pdm_ VLPs were acquired (Figure 3). Upon incubation at 37°C in pH 5·5, cleavage of 3′SL was completed within 2 hours for N1_av_ and N1_pdm_, while N1_sea_ took 12 hours to reach a similar level (Figure 3A). Much weaker cleavage of 6′SL could be observed, and the substrate was not completely hydrolyzed even after 12 hours of incubation, and only 70·4%, 78·1%, and 8·5% of the 6′SL were cleaved by N1_av_, N1_pdm_, and N1_sea_, respectively (Figure 3A). As NA enzymatic activity was inhibited by mild alkaline pH and low temperature, we performed STD-NMR analysis of the NA-VLPs binding with 3′SL or 6′SL in pH 8·5 at 4°C. STD spectra of N1_av_-VLP are shown in Figure 3B, and similar spectra were observed using N1_sea_ and N1_pdm_ (data not shown). The STD-NMR signals for 3′SL were much stronger than those of 6′SL, indicating that NA-VLPs have a preferential binding of α-2,3 sialylglycans compared with their α-2,6 counterpart (Figure 3B).

Despite significant overlap of most of the signals in the region 3·1–3·7 ppm of the spectrum corresponding to the glycan rings, partial analysis on the contribution on the different moieties to the binding (STD-NMR signals) could be performed. Binding of 3′SL to NA-VLP was clearly dominated by the neuraminic acid as all protons in this moiety were detected in STD-NMR spectra (Figure 3B), for example Sia-H3_ax_ at 1·38 ppm, Sia-H3_eq_ at 2·35 ppm, Sia-NHAc at 1·62 ppm, Sia-H7 at 3·18 ppm, and Sia-H9/H9′ at 3·23 ppm and 3·46 ppm. Some contributions from the galactose moiety (Gal-H2 at 3·16 ppm and Gal-H3 at 3·7 ppm) and to a lesser extent from the glucose group (β-Glc-H4 at 3·27 ppm) were also detected in the STD spectra. Signals from other protons were identified in the reference ^1^H-NMR spectrum (e.g., Gal-H1 at 4·12 ppm, Gal-H4 at 3·55 ppm, Gal-H5 and Gal-H6 as multiplet between 3·30 and 3·45 ppm, α/β-Glc-H1 at 4·81/4·25 ppm, and β-Glc-H2 at 2·87 ppm), but these protons showed limited signals in the STD-NMR binding spectra (complete assignment table can be found in Table S1). Although the signal-to-noise ratio in the experiments using 6′SL was not satisfactory for epitope mapping analysis, the terminal sialic acid (Sia) was the major binding group as most of the visible signals in 6′SL binding to NA-VLP were corresponding to the protons of this group.

### Epitope mapping on sialyllactose binding with NA-VLPs

**Figure 4:**
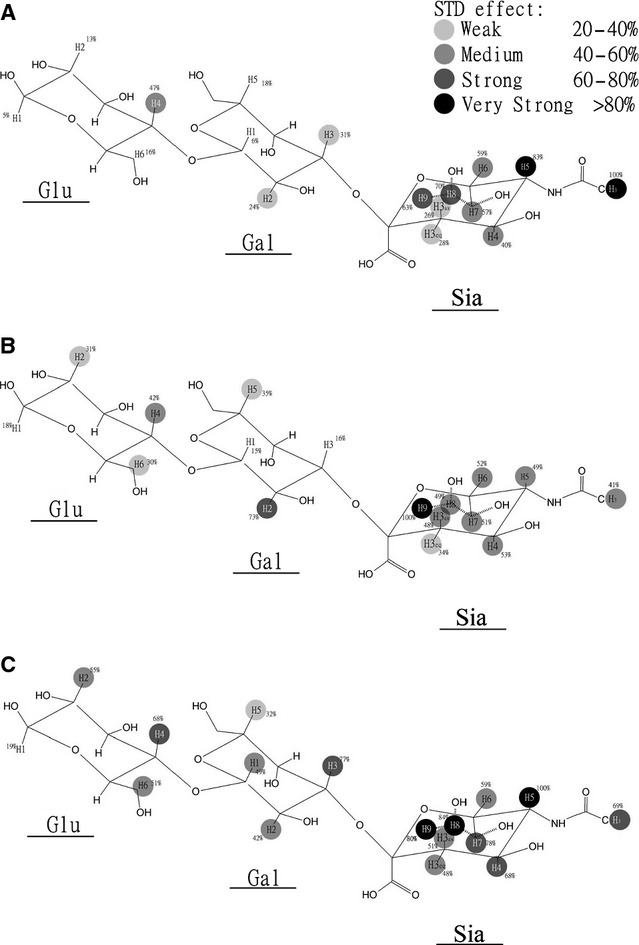
Epitope map of 3′SL with NA-VLPs, of which N1 was derived from avian H5N1 (A), human seasonal (B), or pandemic (C) H1N1 viruses. The strongest STD signal was set to 100% (as indicated in the figure), and relative STD-NMR effects (A_SDT_) were calculated accordingly (percentage on the structures). Difference of saturation transfer difference (D-STD) spectra was calculated by subtraction of STD-NMR spectra acquired for SLs binding to NA-VLP obtained in the presence or in the absence of oseltamivir, respectively.

Sialic acids bind to influenza NA at two independent binding sites: the catalytic site and the secondary binding site. With the catalytic site being blocked by oseltamivir carboxylate, STD-NMR signals of sialyllactoses binding to the secondary site could be evaluated.^17^ Differential STD-NMR spectra were generated by subtracting the secondary site binding signals from the overall NA-SL binding spectra, which reflect only the binding signals of sialyllactoses to the NA catalytic site. Epitope maps were then calculated using STD-NMR effect and are summarized in Figure 4. Epitope mapping was only carried out for 3′SL as the STD effects of 6′SL were too weak for quantitative analysis. As mentioned above, the terminal sialic acid moiety (Sia) was the major binding group to NAs from avian H5N1, human seasonal H1N1, and H1N1pdm09. However, the binding profiles of the three NAs were not the same as shown by comparison of individual atoms in STD-NMR spectra (supplementary material Figure S2). Although the glycerol side chain of the sialic acid (Sia-H7 to Sia-H9) displayed strong STD effect (>60%) in the interactions between 3′SL with all three NAs, STD effect of the acetamido group (NHAc) linked to the Sia-C5 varied from 41% in N1_sea_, 69% in N1_pdm_, to 100% in N1_av_. The STD-NMR signal intensities of the galactose and glucose moieties interacting with NA from different viruses also varied, with less contribution (less STD effects) from the internal sugars in the binding with N1_av_ and N1_sea_ compared with the binding with N1_pdm_. These differences in the STD-NMR effects suggest that the orientation of the ligand in the NA catalytic site may not be the same for NA from different virus origin. Similar finding was reported using *in silico* modeling methods.^18^

### Kinetic assay

**Figure 5:**
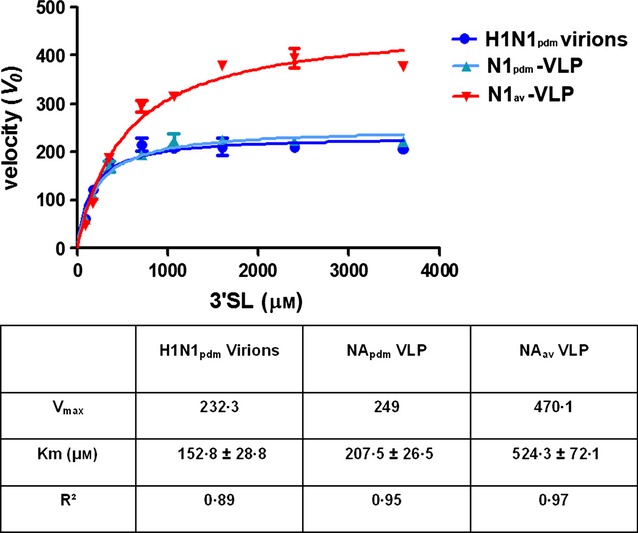
Kinetics of cleavage activity of influenza virus and N1-VLPs on α-2,3-sialyllactose. NA activity of N1_av_-VLP, N1_pdm_-VLP, and H1N1pdm09 native virions was monitored using a galactose oxidase/HRP assay detecting the exposed beta-galactose residue of the products.

We examined the cleavage activity kinetics of neuraminidase derived from NA-VLPs as well as from the native viruses to compare the two NA sources. The cleavage activity was analyzed in an enzymatic assay by monitoring the appearance of fluorogenic product. Kinetic study of NA activity of native virus was restricted to the H1N1pdm09 subtype as highly pathogenic H5N1 subtype requires analysis at the BSL-3 level. Data obtained with the virions and N1-VLPs for the pandemic H1N1 strain are plotted in Figure 5 and show good agreement between the two systems (Km_vlp_ = 207·5 μm, 95% CI: 150·7–264·3 μm; Km_virus_ = 152·8 μm, 95% CI: 91·05–214·5 μm; see table in Figure 5).

## Discussion

In the present work, we have used NMR spectroscopy to monitor (i) the binding of N1 neuraminidases from three strains of influenza (HPAI H5N1, H1N1pdm09, and human seasonal H1N1) and (ii) their sialidase activity. We expressed the N1 on the surface of virus-like particles (VLPs) to maintain their native conformation.^9^ These NA-VLPs mimic the native virions and were analyzed in the absence of HA, while in other reports, the data have been obtained with purified proteins or whole native viruses. This methodology eliminated the possible interference from HA or the alteration of NA folding during the isolation of the purified protein.

Our data indicated that all three N1 enzymatic cleavage activities required an acidic pH (Figures 1A and S1), which is in agreement with previous studies.^19^ The low activity of NA at neutral pH (pH = 7·5) is slightly different from previous findings,^19^,^20^ but could be explained by the use of deuterated buffer in the assay. The actual pH/pD value of the reaction mixture was reported to be higher than the measured pH value by up to 0·4.^21^ The requirement of a slightly acidic pH for efficient NA catalytic activity is in agreement with a physiological condition as reported pH values on the airway surface average about 6·6.^22^ Similar to most enzymes, the kinetics of the sialic acid cleavage at pH 5·5 was significantly inhibited when the reaction mixture was incubated for one hour at 4°C compared with that at 37°C (Figure 1B). Although the temperature dependency of NA activity is not important from a clinical point of view, the reduced activity at low temperature is useful for the study of sialylglycan binding to NA using STD-NMR.

Although the sialidase activity of NA was shown to be pH dependent, it is interesting to note that the effect of pH on NA-ligand binding was found to be a minor component (Figure 2). This is the first experimental evidence showing that an increase in pH inhibited the NA enzymatic activity without significant effect on the ligand recognition. The only observed difference in STD spectra acquired at different pH was the hydrogen atoms linked to the C7 and C9 of neuraminic acid moiety, but whether the difference in STD effect on these two specific positions is related to the lower sialidase activity at higher pH value is not yet defined. It was not appropriate to study the effect of temperature on the NA–Sia binding affinity using our method as an increase in temperature during the STD-NMR experiment would significantly reduce the STD effect.^23^

In the substrate specificity study, all three neuraminidases (N1_av_, N1_sea_, and N1_pdm_) showed a clear preference for cleaving α-2,3 (avian receptor analog) rather than α-2,6 (human receptor analog) sialylglycans (Figure 3A) in agreement with previous reports.^24^–^26^ Binding affinity study using STD-NMR demonstrated that the studied influenza NAs preferentially bind 3′SL rather than 6′SL, which is associated with the stronger activity on the ligand. It is likely that NA substrate specificity is dependent on the binding affinity of NA to different ligands. It will be interesting to perform similar study on an NA with different substrate preference profile, for example with recently isolated N2 subtype viruses which were reported to have similar cleavage on both α-2,3 and α-2,6-linked sialic acids.^27^

Comparison of the enzymatic activity of the different NA showed that N1_av_ and N1_pdm_ had a much stronger activity than N1_sea_ in general and cleaved 6′SL 20–30 times slower than 3′SL. In comparison, for N1_sea_, the cleavage of 6′SL was only about 10 times slower compared with the hydrolysis of 3′SL. A similar substrate preference of the NA from pandemic H1N1 was recently published using recombinant viruses,^28^ and the data obtained with NA-VLPs were very similar to those using native viruses (Figure 5).

It is believed that the binding of HAs (binding avidity and ligand specificity) to host cells has to match the activity of NAs (rate of activity and substrate specificity) for the influenza virus to achieve efficient infection and replication.^27^,^29^ It would be interesting to investigate whether the relatively high activity of N1_av_ and N1_pdm_ correlated with stronger HA-receptor avidity; and the interplay between HA and NA on the same viral surface also needs to be considered. Regarding the specificity study, N1_av_ displayed a substrate preference for 3′SL which matches the preferential binding of the correspondent H5 hemagglutinin to α-2,3-linked sialic acids.^30^ However, N1_sea_ and N1_pdm_ also exhibit 3′SL preference even though the correspondent HAs have weak binding to α-2,3-sialylated glycans,^31^,^32^ suggested a biological importance of the NA to cleave the α-2,3-linked sialic acids (e.g., to remove the decoy receptors).

The STD-NMR method is highlighted for its ability to determine the binding epitope of a ligand binding to its target protein. By subtracting the STD signals of ligand binding to the secondary site from the overall STD spectra of SL interacting with NA, D-STD-NMR spectra were obtained, and epitope maps of 3′SL binding to the catalytic site could be estimated. It appears that the main contribution to the NA binding came from Sia residues [Sia-NHAc (1·62 ppm), Sia-H3_eq_ (2·35 ppm), and Sia-H7, Sia-H8, Sia-H9 from glycerol side chain of Sia (3·18–3·48 ppm)] rather than the internal sugars, although these were still involved to a minor extent (Figure 4). Saturation transfer to the protons of galactose was significantly less effective, and for the reducing glucose residue, very limited STD signal was detected (except for N1_sea_). Although the binding profiles of the three NAs with 3′SL looked similar in general, detailed STD effects of specific positions were not the same as shown by the comparison of individual atoms in STD spectra (Figure S2). For example, despite the C6 glycerol side chain showing a high STD signal in binding with all three N1-VLPs, STD signals of the acetamido group (NHAc) linked to the Sia-C5 varied from a preponderance signal in binding with N1_av_ to a strong STD effect with N1_pdm_ and to an even further reduced effect in the binding with N1_sea_. Subtle differences in the STD signal intensities of the galactose and glucose moieties interacting with NA from different viruses were also observed especially in N1_sea_, which showed significantly higher STD effects from the inner sugars (Figure 4). This finding could explain the difference in NA activity of different virus strains; for example, stronger binding of the NHAc moiety in N1_av_ and N1_pdm_ may be responsible for the overall higher enzymatic activities. A recent article by Xu and colleagues has shown different findings, but as these researchers used soluble recombinant NA, the comparison with our NA-containing VLPs system cannot be carried out.^33^ However, further studies on the impact on the difference in the systems used are necessary to examine this hypothesis. It is important to note that the contribution of inner sugars in sialylglycans on the interaction with NA was not available from X-ray crystallography studies as most ligands were unitized by NA during co-crystallization. Therefore, the epitope mapping data at atomic resolution produced by STD-NMR could be very useful in the study of NA–ligand interaction. In fact, our findings are the first reported experimental data on the binding epitope of sialylglycan interacting with NA including the internal sugars, and the finding is in agreement with previous report using *in silico* modeling.^18^ However, the presented study has the limitations on the ligand availability and ligand size. Specific ligands that represent the actual sialylated glycans in the human respiratory tract^34^ are necessary to increase the biological relevance. Further development of the methodology, such as multidimensional STD-NMR, will also be required for the study on more complex ligands.

In conclusion, the data presented in this study demonstrated that physicochemical conditions (pH in particular) could affect the NA enzymatic activity with minor effect on ligand binding. The low activity at 4°C and pH 8·5 allowed us to investigate the binding affinity of the different sialosides with NA from different strains without interference by the catalytic hydrolysis of the ligands. NA cleavage specificity seemed to be associated with a difference in binding affinity to different ligands, suggesting a relationship between the two events. It is possible that NA activity on different substrates is dependent on the binding of NA to those ligands. These findings have implications regarding the replication cycle of influenza infection in the host where different sialidase activities would influence penetration through the respiratory mucin barrier and the release of the newly generated virus from the infected cells. Additional investigation on the physiological pH in respiratory tract, as well as the precise glycan compositions determining cleavage activity will thus lead to a more comprehensive understanding on the precise role of the NA and the balance between its two functions (binding and cleavage).

## Supporting Information

**Figure S1.** Effect of pH on cleavage activity of N1pdm and N1sea.

**Figure S2.** STD NMR spectra of α-2,3-sialyllactose binding to different NA-VLP.

**Table S1.** Chemical shift of hydrogen atom in sialyllactoses.
