# Supplementary figures and images for: Investigation of the binding and cleavage characteristics of N1 neuraminidases from avian, seasonal, and pandemic influenza viruses using saturation transfer difference nuclear magnetic resonance
Source: Influenza Other Respir Viruses. 2013 Sep 30;8(2):235–42. doi: 10.1111/irv.12184 (PMC4186472; doi:10.1111/irv.12184)

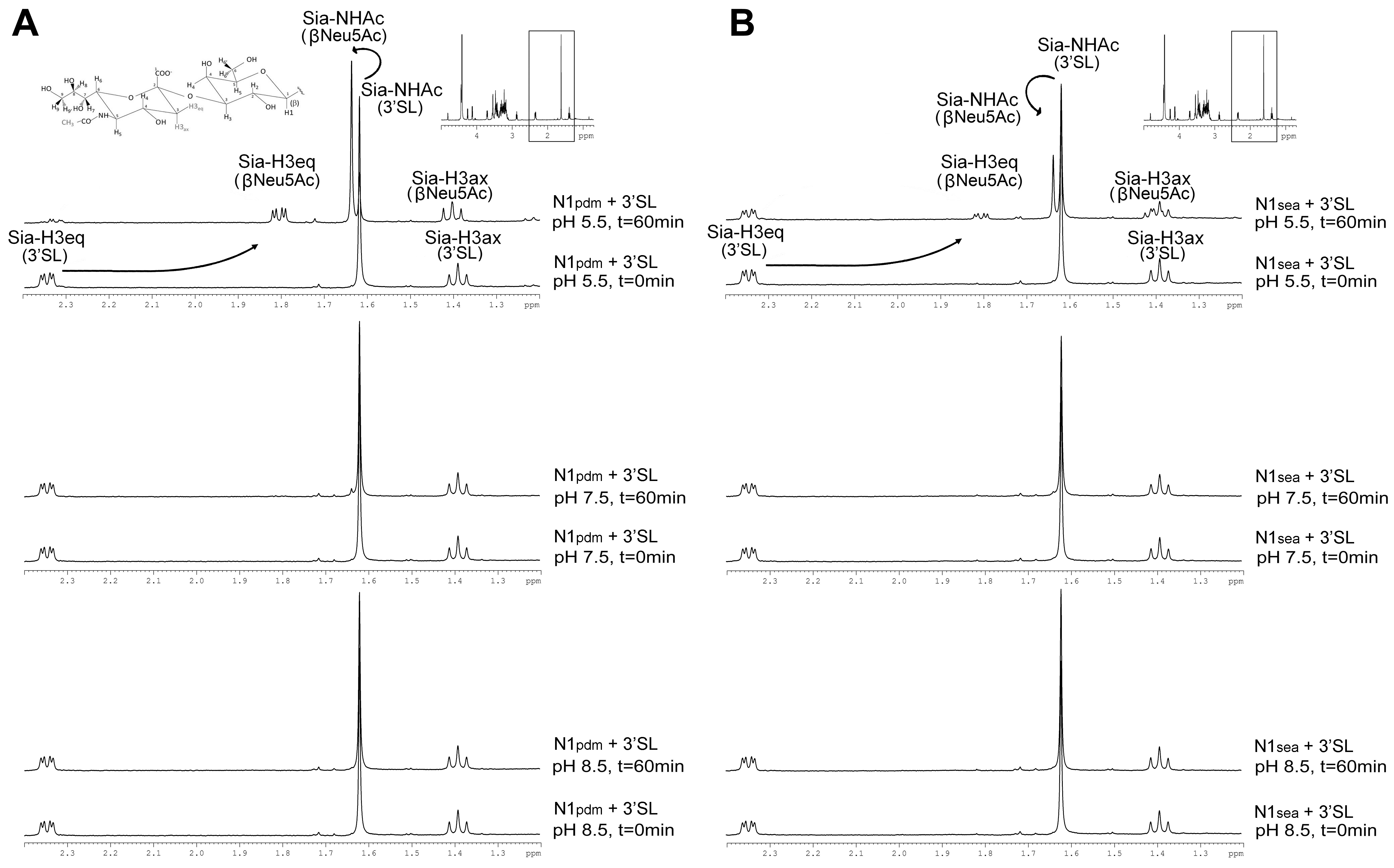

Supplement: Supplementary file 1 — Figure S1. Effect of pH on cleavage activity of N1pdm and N1sea. [file irv0008-0235-SD1.tif]

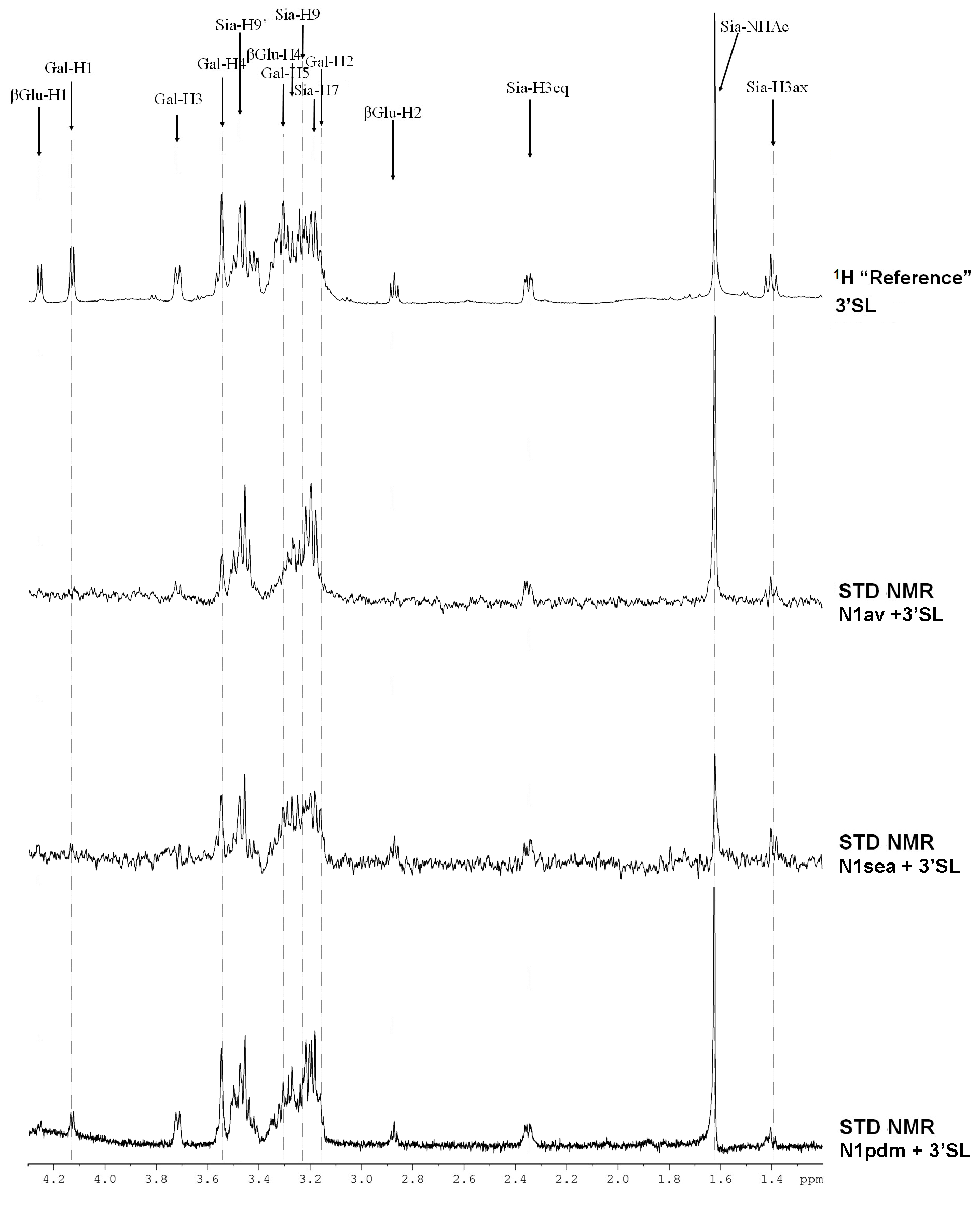

Supplement: Supplementary file 2 — Figure S2. STD NMR spectra of α-2,3-sialyllactose binding to different NA-VLP. [file irv0008-0235-SD2.tif]

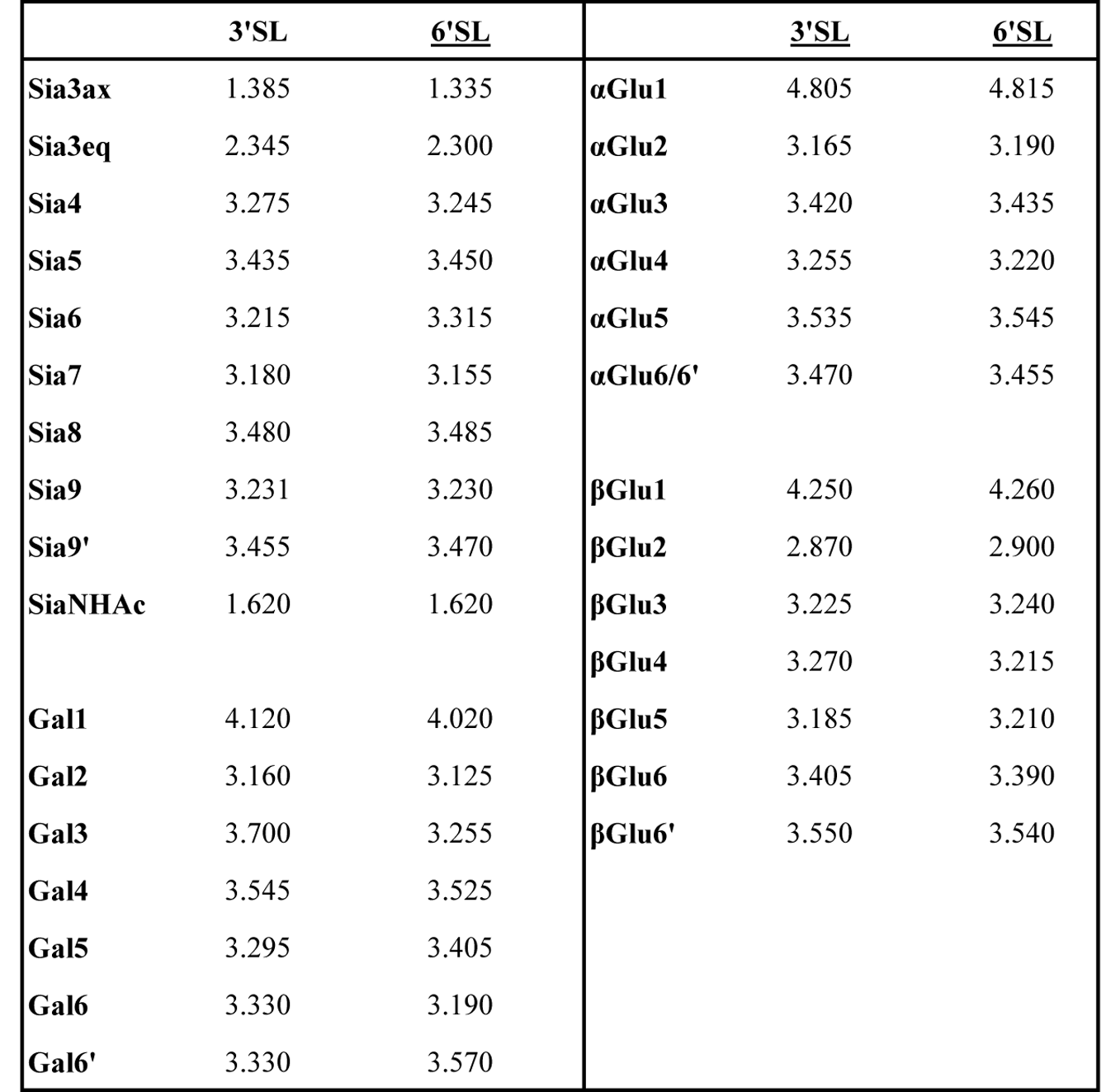

Supplement: Supplementary file 3 — Table S1. Chemical shift of hydrogen atom in sialyllactoses. [file irv0008-0235-SD3.tif]
