# Supplementary material for: Investigation of the binding and cleavage characteristics of N1 neuraminidases from avian, seasonal, and pandemic influenza viruses using saturation transfer difference nuclear magnetic resonance
Source: Influenza Other Respir Viruses. 2013 Sep 30;8(2):235–42. doi: 10.1111/irv.12184 (PMC4186472; doi:10.1111/irv.12184)
Supplement: Supplementary file 4 — Data S1. Materials and Methods. [file irv0008-0235-SD4.docx]

Supplementary Materials

**Supplementary Information:**

**Figure S1. Effect of pH on cleavage activity of N1pdm and N1sea.** NA-VLP from human H1N1pdm09 (A) and seasonal H1N1 (B) were incubated at different pH with α-2,3-sialyllactose. The cleavage reaction was monitored by ^1^H NMR over 1 hour.

**Figure S2. STD NMR spectra of α-2,3-sialyllactose binding to different NA-VLP.**

**Table S1. Chemical shift of hydrogen atom in sialyllactoses.**

**Materials and Methods**

*Ligands and other chemicals*

All chemicals, except otherwise stated, were purchased from Sigma-Aldrich with the highest purity available. α-2,3- and α-2,6-sialyllactose (3’SL and 6’SL), were used as ligands for the avian and human receptors respectively, and were synthesised as reported ([1](#_ENREF_1)). All NMR experiments were performed on a Bruker Avance 600MHz spectrometer, equipped with a 5-mm TXI probe with triple axis gradients. Peak assignments of ligands were obtained from classical proton-carbon HSQC and proton-proton TOCSY NMR experiments (See table S1 in supplementary material) correlated by data published by Pan *et al*. ([2](#_ENREF_2))).

*Production and purification of NA-VLP and viruses*

Virus-like particles containing FLAG-tagged NA of influenza A/Cambodia/JP52a/2005 (H5N1), A/Gansu/Chenguan/1129/2007 (human seasonal H1N1) and A/California/04/2009 (H1N1pdm09) were produced and analysed as previously described ([3](#_ENREF_3)). Briefly, HEK-293T cells were transfected with the representative flagged-N1 gene and cloned into the pcDNA-3.1 vector (Invitrogen). Culture medium was collected and filtered, followed by ultracentrifugation using a Beckman SW32 rotor at 4°C with a speed of 28,000 rpm for 2h30 with a 30% sucrose-HEPES cushion (2 mM HEPES, 125 mM NaCl, 0.9 mM CaCl_2_, 0.5 mM MgCl_2_, pH=7.4). The NA-VLP pellet was then resuspended in HEPES buffer and stored in -80°C. NA content in the VLP was quantified using western blotting with an Anti-FLAG monoclonal mouse antibody (Sigma). NA enzymatic activity was checked with NA-Star chemoluminescent assay according to manufacturer’s instructions (Applied Biosystems). Influenza A/California/04/2009 (H1N1) virus was propagated in MDCK cells with culture medium supplemented with 0.5 mg/mL phenylalanyl chloromethyl ketone-treated trypsin.

*STD NMR evaluation of binding activity*

STD NMR spectra were acquired at 600 MHz and 279 K. NA-VLPs were diluted to 8 μM of catalytic sites in 250 μL of HEPES buffer for each experiment. Sample buffer was exchanged three times using Amicon (100 kDa cut-off, Millipore) against HBS-d18 (2 mM HEPES-d18, 125 mM NaCl, 0.9 mM CaCl_2_, 0.5 mM MgCl_2_, in D_2_O) at 4°C, with centrifugation at 4,000 rcf. The protein was saturated ON-resonance at -1 ppm and OFF-resonance at 300 ppm with a cascade of 60 selective Gaussian-shaped pulses of 50 ms duration. A 100 μs delay between each pulse was applied, resulting in a total saturation time of 3 s and a relaxation delay of 4 s was used. A total of 1,024 scans per STD NMR experiment were acquired and a WATERGATE sequence was used to suppress the residual HDO signal. A spin-lock filter was applied to suppress protein background. 3’SL or 6’SL was added to give a molecular protein:ligand ratio of 1:100. ON- and OFF-resonance spectra were stored and processed separately, and the final STD NMR spectra were obtained by subtracting the ON- and OFF-resonance spectra. Control STD NMR experiments were performed with an identical setup but in the absence of protein or ligand. In order to remove the binding signals of the hemadsorption site from that of the catalytic site, a difference of STD NMR spectra were obtained by subtraction between STD NMR spectra obtained in the presence of oseltamivir carboxylate (blocking the enzymatic site as previously reported ([4](#_ENREF_4))) with the STD NMR spectra in the absence of the drug.

*NMR experiments for the detection of NA enzymatic activity*

NA-VLPs were diluted to 8 μM of catalytic sites in HBS-d18 as described above. The apparent pH of the buffer, ranging from 5.5 to 8.5, was adjusted with 1M NaOH. Either 3’SL or 6’SL was added to the VLP samples to a concentration of 800 μM (molecular protein to ligand ratio of 1:100) and incubated at 4°C or 37°C. ^1^H NMR spectra were measured, before and after incubation with the ligand, at 298 K upon different incubation time, acquired with 32 scans with 2 s relaxation delay over a spectral width of 6,000 Hz.

*Interaction epitope mapping*

The relative STD NMR effects were calculated according to the formula ([5](#_ENREF_5)): A_STD_ = (I_0_ x I_sat_)/I_0_ = I_STD_/I_0_. The proton with highest STD effect was set to 100% and relative STD effects of other protons were calculated accordingly.

*Enzymatic kinetic assay*

Sialidase activity was assessed using an enzymatic assay as described previously in ([6](#_ENREF_6)). Briefly, VLP or influenza virus was incubated with 3’SL (final concentrations from 0 to 3,600 µM) at pH=6.0 with 0.2 M phosphate buffer, excess galactose oxidase (Sigma-Aldrich), horseradish peroxidase (Invitrogen), and Amplex UltraRed reagent (Invitrogen). The reaction was conducted at 37°C and the fluorogenic product was measured every 5 min for 2 h using FLUOstar OPTIMA (BMG Labtech), with excitation and emission wavelengths of 530 and 590 nm, respectively. The enzyme kinetics data were calculated by using nonlinear regression (GraphPad Prism) to determine the Michaelis constant (*K*_M_) and maximum velocity (*V*_max_) of substrate conversion.

**References of Material Methods section:**

1. Kiefel MJ, Beisner B, Bennett S, Holmes ID, von Itzstein M. Synthesis and biological evaluation of N-acetylneuraminic acid-based rotavirus inhibitors. J Med Chem. 1996 Mar 15;39(6):1314-20. PubMed PMID: 8632438. Epub 1996/03/15. eng.

2. Pan GG, Melton LD. Lactones of disialyl lactose: characterisation by NMR and mass spectra. Carbohydr Res. 2006 May 1;341(6):730-7. Epub 2006/02/07. eng.

3. Lai JC, Chan WW, Kien F, Nicholls JM, Peiris JS, Garcia JM. Formation of virus-like particles from human cell lines exclusively expressing influenza neuraminidase. J Gen Virol. 2010 Sep;91(Pt 9):2322-30. PubMed PMID: 20505010. Epub 2010/05/28. eng.

4. Lai JC, Garcia JM, Dyason JC, Bohm R, Madge PD, Rose FJ, et al. A secondary sialic acid binding site on influenza virus neuraminidase: fact or fiction? Angew Chem Int Ed Engl. 2012 Feb 27;51(9):2221-4. PubMed PMID: 22281708. Epub 2012/01/28. eng.

5. Mayer M, Meyer B. Group epitope mapping by saturation transfer difference NMR to identify segments of a ligand in direct contact with a protein receptor. J Am Chem Soc. 2001 Jun 27;123(25):6108-17. PubMed PMID: 11414845. Epub 2001/06/21. eng.

6. Yen HL, Liang CH, Wu CY, Forrest HL, Ferguson A, Choy KT, et al. Hemagglutinin-neuraminidase balance confers respiratory-droplet transmissibility of the pandemic H1N1 influenza virus in ferrets. Proc Natl Acad Sci U S A. 2011 Aug 23;108(34):14264-9. PubMed PMID: 21825167. Pubmed Central PMCID: 3161546. Epub 2011/08/10. eng.
